# Patterns of Genetic and Morphological Variability of *Teucrium montanum sensu lato* (Lamiaceae) on the Balkan Peninsula

**DOI:** 10.3390/plants13243596

**Published:** 2024-12-23

**Authors:** Miloš Zbiljić, Dmitar Lakušić, Zlatko Šatović, Zlatko Liber, Nevena Kuzmanović

**Affiliations:** 1 Department of Botany, Faculty of Pharmacy, University of Belgrade, Vojvode Stepe 450, 11060 Belgrade, Serbia; 2 Institute of Botany and Botanical Garden, Faculty of Biology, University of Belgrade, Takovska 43, 11000 Belgrade, Serbia; dlakusic@bio.bg.ac.rs (D.L.); nkuzmanovic@bio.bg.ac.rs (N.K.); 3 Department of Plant Biodiversity, Faculty of Agriculture, University of Zagreb, Svetošimunska 25, 10000 Zagreb, Croatia; zsatovic@agr.hr; 4 Centre of Excellence for Biodiversity and Molecular Plant Breeding (CroP-BioDiv), Svetošimunska 25, 10000 Zagreb, Croatia; zlatko.liber@biol.pmf.hr; 5 Division of Botany, Department of Biology, Faculty of Science, University of Zagreb, Marulićev trg 9A, 10000 Zagreb, Croatia

**Keywords:** AFLP, Balkans, BAPS, *Teucrium montanum*, K-means, morphometry, genetic diversification

## Abstract

The Balkan Peninsula represents an important center of plant diversity, exhibiting remarkable ecological heterogeneity that renders it an optimal region for studying the diversification patterns of complex taxa such as *Teucrium montanum*. In the Balkan Peninsula, *T. montanum* is a highly plastic and morphologically variable species with unresolved taxonomic status. To ascertain the patterns of genetic and morphological diversification, a comparative genetic and morphological analysis was conducted. In total, 57 populations were subjected to analysis using AFLP and a multivariate morphometric approach. A Bayesian analysis of population structure distinguished two main genetic clusters, labelled A and B. Cluster B was found to be geographically restricted to the northwestern Dinarides, while cluster A occurred in the rest of the Balkans. Genetic cluster A was further subdivided into four subclusters that were spatially separated from each other. The contact populations between the subclusters exhibited a mixed genetic structure. There was a partial correlation between genetic and morphological diversification. The peripheral populations of the genetic clusters displayed morphological differences, while both genetic and morphological differences decreased in the contact zones. The observed genetic structure can be attributed to the reproductive biology of this species and the complex geological history of the Balkan Peninsula.

## 1. Introduction

The Balkan Peninsula, characterized by complex geological and climatic heterogeneity, represents a critical biodiversity hotspot in Southeast Europe [1,2,3] and serves as an important glacial refuge for plant species [3]. Moreover, the region harbors a significant number of endemic and relict species [4,5,6]. The considerable geographical heterogeneity combined with a mosaic of different habitats and long-lasting ecological stability have facilitated the diversification of evolutionary lineages and promoted the long-term survival of a large number of species. Therefore, the Balkan Peninsula offers a unique opportunity to investigate both historical and ongoing evolutionary processes that underlie the complex phylogeographic patterns observed in plant species [3].

*Teucrium montanum* L. is a highly polymorphic [7,8,9] semi-woody dwarf shrub that is widely distributed across Europe, Asia Minor and Algeria in North Africa [10]. It typically grows in open, rocky habitats in mountainous regions [11], but it can also be found in a range of habitats, from rocky coastal areas to meadows, cliffs and screes in subalpine and alpine belts [8,9]. Morphologically, it is highly variable [9,11] and plastic [7,12], which has resulted in the description of numerous taxa that are currently treated as synonyms of *Teucrium montanum sensu lato* [13,14]. Over time, there has been no scientific consensus on the status of the described taxa [15], resulting in taxonomic differences in regional floras [1,10,16,17,18]. A comprehensive morphological study has revealed that this species is represented in the Balkans by seven distinct morphological groups (“skadarensis”, “montanum”, “pannonicum”, “skorpili”, “luteolum”, “helianthemoides” and “parnassicum”), which can be easily distinguished by specific combinations of morphological traits [9]. The distribution of morphological groups only partially corresponds to ecological diversification [9]. Despite the distinct morphological characteristics of certain populations, most of the taxa described in the Balkans are regarded as synonyms of *T. montanum*. Currently, only *T. montanum* subsp. *helianthemoides* (Adamović) Baden is recognized as an accepted taxon from this region [13,14]. As five morphological groups correspond to previously described taxa at specific or intraspecific levels, and two have a specific combination of morphological characters that have not been described before [9], it was important to perform genetic analyses on these populations in order to elucidate their phylogeographic relationships. Specifically, our main aims were (a) to examine the genetic diversity, population structure, and differentiation; (b) to investigate the patterns of genetic diversification; and (c) to ascertain the extent of overlap between genetic and morphological clustering of individuals.

## 2. Results

### 2.1. Genetic Diversification of Teucrium montanum sensu lato on the Balkan Peninsula

A total of 490 polymorphic AFLP markers were identified in 260 individuals. The global mismatch error rate for all four selective PCR primer combinations was 0.403% [19].

The proportion of polymorphic loci found (*P%*) was between 0.104 and 0.255, with a mean value of 0.157. The highest proportions were observed in populations P09 AL-Tomorit (0.214), P12 HR-Bisko, Trilj (0.202), P20 HR-Lanišće (0.255), and P22 BH-Trebinje (0.202). The lowest values were observed in populations P02 GR-Eubea Dirfi (0.124), P21 HR-Žumberak (0.122), P37 SR-Orovica (0.104), P45 GR-Chianochori (0.114) and in the species *T. capitatum* L. with 0.206 (Table S1). The number of private alleles (*N_pr_*) varied between zero and four, with 38 populations having no private alleles. One private allele was detected in nine populations (P03 GR-Ossa, P06 MA-Matka Canyon, P08 GR-Smolikas, P11 HR-Biokovo, P22 BH-Trebinje, P32 SR-Šarplanina, Piribeg, P42 AL-Ostrovica, P45 GR-Chianochori, and P47 SR-Gornjak), two private alleles were detected in three populations (P01 MA-Ohrid, P37 MA-Orovica, and P39 RU-Nera Canyon), and four private alleles were found in one population (P13 HR-Murter). The Shannon index (*I*) varied between 0.085 and 0.199, with a mean value of 0.127. The highest values were observed in populations P09 AL-Tomorit (0.159), P12 HR-Bisko, Trilj (0.159), P20 HR-Lanišće (0.199), P22 BH-Trebinje (0.163), P32 AL-Šarplanina, Pribeg (0.155), and P50 AL-Skadar (0.156), while the lowest values were observed in populations P02 GR-Dirfi (0.101), P06 MA-Matka Canyon (0.100), P21 HR-Žumberak (0.099), P37 SR-Orovica (0.085), P45 GR-Chianochori (0.093) and in the species *T. capitatum* with 0.170 (Table S1). The expected heterozygosity (*H_E_*) ranged from 0.059 to 0.093, with a mean value of 0.075. The highest values were recorded in populations P12 HR-Bisko, Trilj (0.089), P13 HR-Murter (0.088), P14 HR-Gračac (0.087), P20 HR-Lanišće (0.093), and P22 BH-Trebinje (0.088), while the lowest values were recorded in populations P02 GR-Dirfi (0.066), P06 MA-Matka Canyon (0.065), P21 HR-Žumberak (0.062), P37 SR-Orovica (0.059), P44 GR-Kryoneri (0.066), P45 GR-Chianochori (0.061), and in the species *T. capitatum* with 0.105 (Table S1).

**Figure 1:**
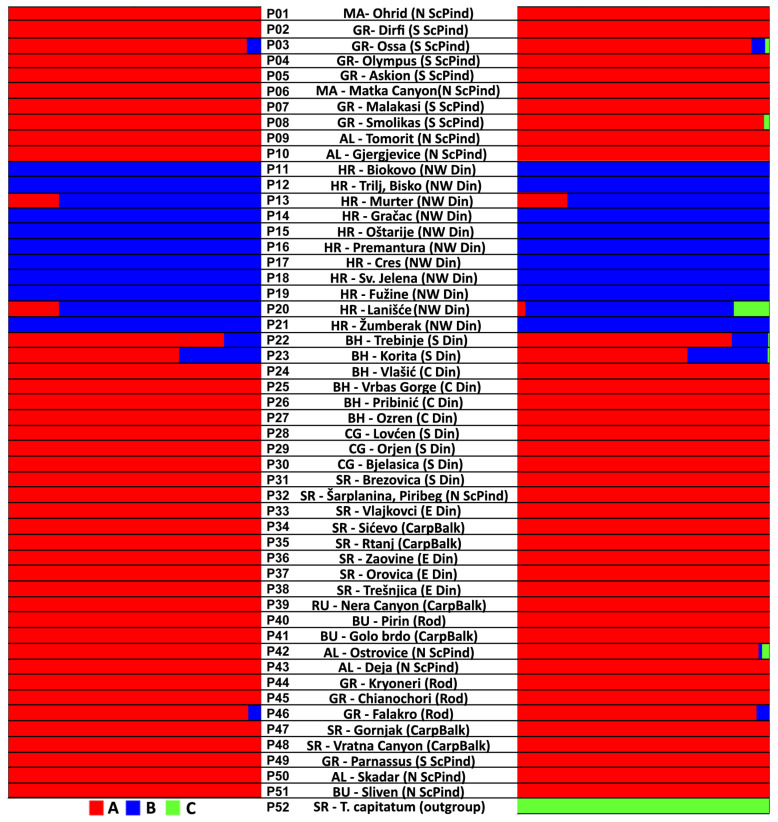
Genetic clusters (groups) obtained from BAPS analysis without an outgroup (left chart) and with the outgroup *T. capitatum* from the Sićevo locality (right chart). Abbreviations: MA—North Macedonian, GR – Greece, AL—Albania, HR—Croatia, BH—Bosnia and Herzegovina, CG—Montenegro, SR—Serbia, RU—Romania, BU—Bulgaria. Din—Dinarides, ScPind—Scardo-Pindhic mountain system, CarpBalk—Carpathian-Balkan mountain system, Rod—Rhodopes.

The genetic structure of the populations was analyzed using the Bayesian approach to population structure analysis in the BAPS v. 6.0 software. The genetic structure analysis with BAPS was performed twice, both with and without an outgroup. The first analysis, conducted without the outgroup, revealed that the analyzed individuals from 51 populations were categorized into two genetic clusters, A and B (Figure 1, Table S1). The genetic structure of the analyzed populations indicates that the majority of individuals within the populations belong exclusively to one of the two genetic clusters. Only a few individuals from populations P03 GR-Ossa, P13 HR-Murter, P20 HR-Lanišće, P22 BH-Trebinje, P23 BH-Korita and P46 GR-Falakro exhibited a certain degree of admixture, although they predominantly belonged to a single genetic cluster. As anticipated, the second analysis, which also included the population of *T. capitatum*, revealed three genetic clusters (Figure 1, Table S1), with *T. capitatum* belonging entirely to genetic cluster C (Figure 1, Table S1). Furthermore, a minor proportion of the genome originating from genetic cluster C was detected in six individuals exhibiting a mixed A-B-C genetic structure from the populations P03 GR-Ossa, P20 HR-Lanišće, P22 BH-Trebinje, P23 BH-Korita and P42 AL-Ostrovica. In addition, a mixed A-C genetic structure was observed in one individual from population P42 AL-Ostrovica and one individual from population P08 GR-Smolikas. With the exception of one individual from population P20 (HR-Lanišće), in which the proportion of cluster C reached 70%, the proportion of cluster C in the remaining mixed individuals varied between 1% and 14%.

**Figure 2:**
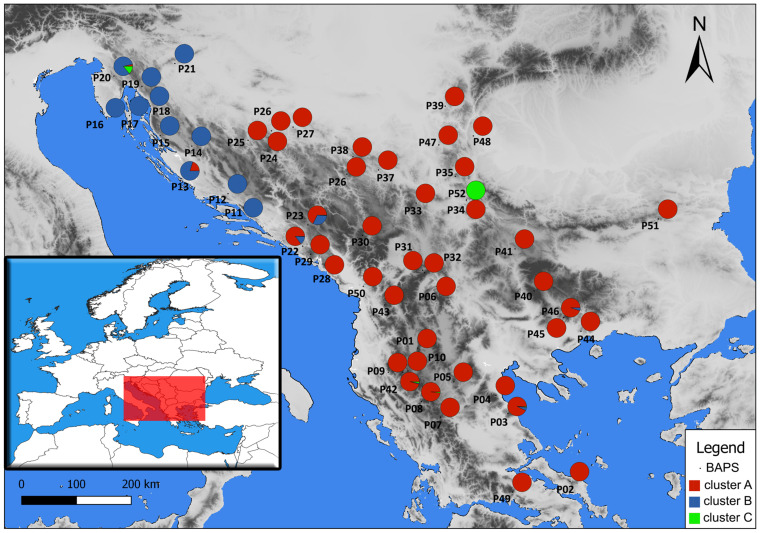
Distribution of the analyzed populations with labels indicating their affiliation to genetic clusters based on the BAPS analysis of the population genetic structure with an outgroup in the studied area.

There is a clear spatial separation between genetic clusters A and B. Genetic cluster B (*T. montanum* s. str.) is geographically confined to the extreme northwest of the Balkan Peninsula, encompassing populations in the coastal and island regions of central and northern Adriatic Croatia, the continental part of the Istrian Peninsula, the Lika region and Mount Žumberak. Genetic cluster A (*T. montanum* s.l.) is distributed across other parts of the Balkan Peninsula (Figure 2).

**Figure 3:**
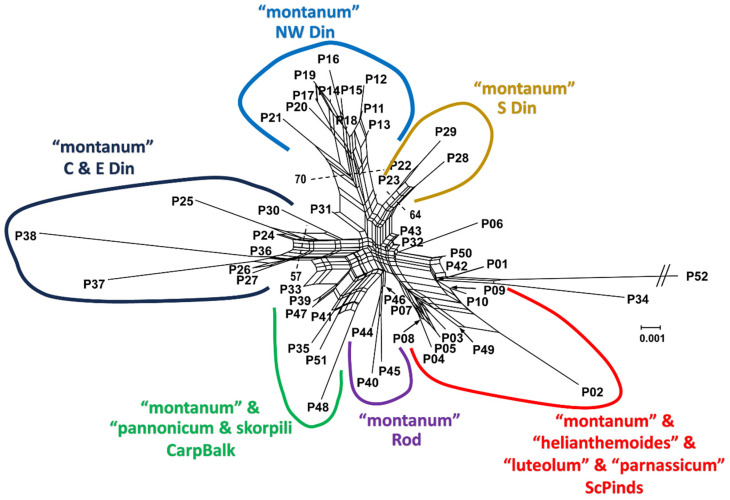
The neighbor-net diagram based on Nei’s distance matrix of *T. montanum* and *T. capitatum* populations (pop 52, genetic cluster C—capitatum). Bootstrap values exceeding 50% are indicated near the branches. The diagram illustrates the population affiliation to the morphological groups and mountain range according to the classification proposed by Zbiljić et al. [9]. The following abbreviations are used: Din—Dinarides, CarpBalk—Carpathian-Balkan mountains, Rod—Rhodopes, ScPinds—Scardo-Pindhic mountains.

Only three groups of populations exhibited a distinct position in the phylogenetic network with a bootstrap support of more than 50% (Figure 3). Despite the fact that these groups were distributed across different parts of the Dinaric Mountain system and exhibit genetic divergence, all populations belong to the morphological group “montanum”. The separation of populations (P11–P21 from Croatia) from the northwestern Dinarides showed the highest degree of bootstrap support (70%) and fully corresponded to genetic cluster B identified in BAPS. This was followed by the group of populations from the southern Dinarides—P22 and P23 from Bosnia and Herzegovina, and P28 and P29 from the Montenegrin coast—that exhibited a bootstrap support of 64%. Finally, the populations from the central and eastern parts of the Dinaric Mountain system (P24, P25, P26, P27 from Bosnia and Herzegovina and P37 and P38 from western Serbia) exhibited the lowest bootstrap support (57%). Despite the lack of statistical support for the separation of other genetic groups, the populations occurring on the same mountain range were situated in the same part of the phylogenetic network. Thus, the populations from the Carpathian-Balkan massif (P33, P35 and P48 from Serbia, P39 from southwestern Romania, P41 and P51 from Bulgaria) were situated in the same part of the network and constituted a combination of populations from three morphological groups: “montanum”, “pannonicum” and “skorpili”. The group of populations from the Rhodope Mountains (P40 from Pirin, P44, P45 and P46 from north-eastern Greece) were located in a separate part of the phylogenetic network and exhibited a uniform morphological identity, belonging to the morphological group “montanum”. In contrast, the group of populations from the Scardo-Pindhic mountain system (P1 from North Macedonia, P3, P4, P5, P7, P8 from North Central Greece, P9, P10 and P42 from Albania, P49 and P2 from southern Greece) was distinguished as a discrete cluster within the network. However, the bootstrap support for this separation is less than 50%, and the group is morphologically the most diverse, including even four morphological groups (“montanum”, “luteolum”, “helianthemoides” and “parnassicum”) (Figure 3). In the phylogenetic network, a limited number of populations were positioned outside the previously mentioned groups. Populations P32 SR-Šarplanina, Pribeg, P42 AL-Ostrovice, P43 AL-Deja, P06 MA-Matka Canyon and P50 AL-Skadar were situated in the central part of the network, between the southern Dinaric group and the outgroup *T. capitatum* and *T. montanum* from SR-Sićevo. In addition, population P33 SR-Vlajkovci was positioned at the transition between the groups from the central and eastern Dinarides and the Carpathian-Balkan massif. Populations P30 CG-Bjelasica and P31 SR-Brezovica were positioned at the boundary between the groups representing the central and eastern Dinarides and those pertaining to the northwestern Dinarides (Figure 3).

**Figure 4:**
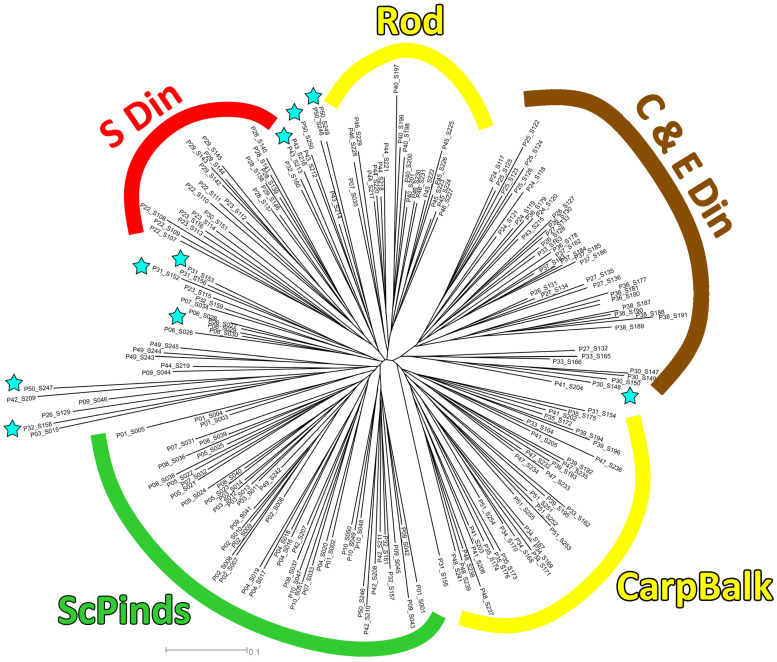
Neighbor-joining tree based on Jaccard distances among individuals; brown, red, green and yellow lines indicate four clusters inferred by non-hierarchical K-means clustering. Population numbers correspond to Table S1 and Figure 1. Admixed populations identified by K-means clustering are marked with light blue asterisks and in the Table S1 with asterisks (*). The following abbreviations are used: Din—Dinarides, CarpBalk—Carpathian-Balkan mountains, Rod—Rhodopes, ScPinds—Scardo-Pindhic mountains.

**Figure 5:**
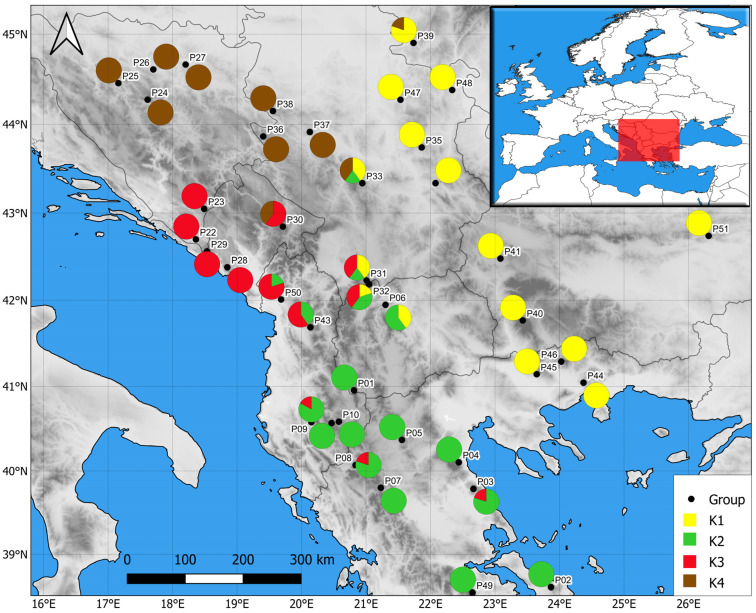
Geographic distribution of the population with labels indicating their affiliation to genetic subclusters identified by K-means clustering. Population numbers correspond to Table S1, Figure 1 and Figure 4.

In order to ascertain the fine relationships between populations within genetic cluster A, neighbor-joining diagram and non-hierarchical K-means clustering was applied exclusively to the populations belonging to genetic cluster A. The neighbor-joining diagram revealed the deepest split between the central and eastern Dinaric group of populations and the Carpatho-Balkan and Rhodope group (Figure 4). Moreover, the latter group was subdivided into the Carpatho-Balkan (P34, P35, P39, P41, P47, P48, P51) and Rhodope groups of populations (P40, P44, P45, P46). Non-hierarchical K-means clustering revealed an optimal separation of the populations into four geographically defined subclusters (central and eastern Dinaric group, southern Dinaric group, Carpatho-Balkan and Rhodope group, and Scardo-Pindhic group; Figure 5). Most populations were assigned to a single subcluster, particularly the populations from the central and eastern parts of the Dinaric mountain massif (P24, P25, P26, P27, P36, P37, and P38), and populations from the eastern part of the Balkan Peninsula (Carpatho-Balkan and Rhodope massifs). The exception in the second group is the P39 Nera Canyon population that was partly assigned to the Dinaric group (Figure 5). Most of the populations geographically positioned between the southern Dinaric and Scardo-Pindhic groups were admixed, namely the populations P06, P30, P31, P32, P43 and P50 (Figure 5).

### 2.2. Morphological Diversification of Genetic Groups

**Figure 6:**
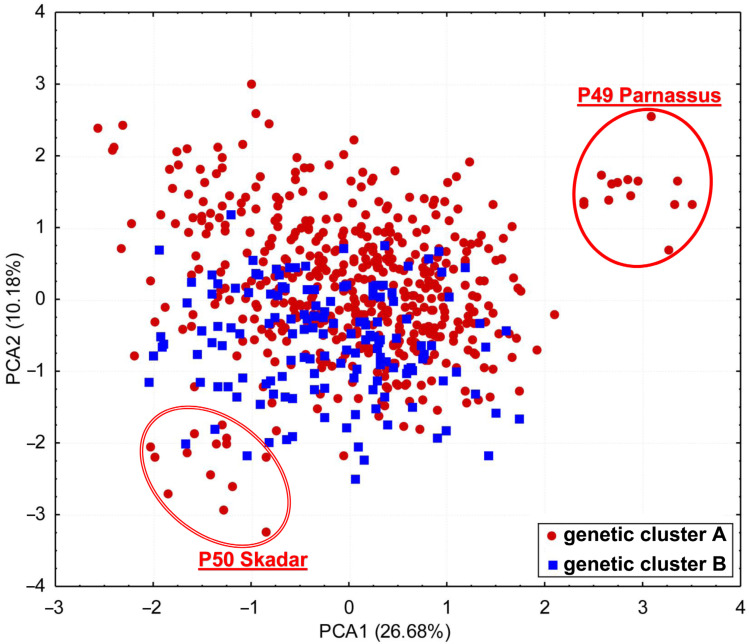
Scatterplot of principal component analysis (PCA), conducted based on 25 morphological characters for two genetic clusters (A and B), defined by the “BAPS” analysis.

Principal component analysis (PCA) was conducted on the basis of 25 morphological characters (Table S2), which were identified as the most significant contributors to the observed variability. The analysis included individuals from 51 populations that were part of the AFLPs. The first two axes cumulatively accounted for 36.86% of the total variability, with the first axis accounting for 26.68% and the second axis contributing an additional 10.18%. In the space defined by the first and second PCA axes, there was an overlap of individuals belonging to groups A and B identified in the BAPS analysis (Figure 5). Nonetheless, populations P49 GR-Parnassus (morphological group “parnasicum”) and P50 AL-Skadar (morphological group “skadarensis”) were to some extent separated from others in the space of the first two axes. The P49 GR-Parnassus population was completely separated from the other individuals and was located in the positive part of the ordination space for both the first and second axes, while the P50 AL-Skadar population was positioned in the negative part of the ordination space for both the first and second axes (Figure 6).

**Figure 7:**
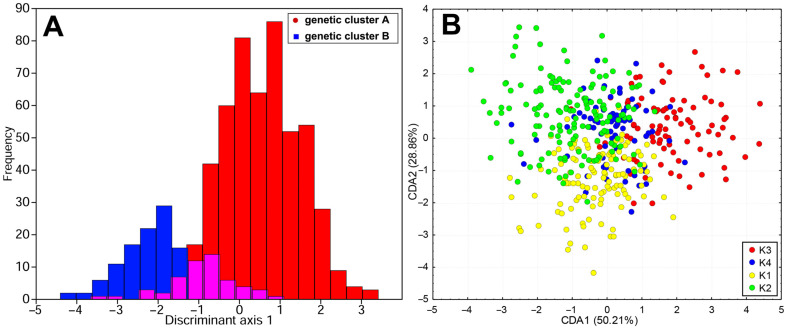
Canonical discriminant analysis (CDA) conducted based on 31 morphological characters: (**A**)—CDA histograms including two genetic clusters (A and B) defined by the “BAPS” analysis; (**B**)—CDA scatterplot including populations classified into four *a priori* groups inferred by non-hierarchical *K*-means clustering. The purple bars represent the overlapping zone of the blue and red bars.

The CDA histogram, which includes two genetic groups of *T*. *montanum* obtained in BAPS (A and B), revealed partial separation along the first axis. The characters that contributed most to the observed discrimination between genetic groups A and B were leaf curvature, radius of the oil-containing cell, bract length, and distance between the calyx base and tooth base (Figure 7A). The first and second CDA axes from the CDA scatterplot were found to account for 79.07% of the observed discrimination. The CDA showed a partial separation of groups K1, K2 and K3 (Figure 7B), while the remaining group K4 occupied a central position in the space defined by the first and second discriminant axes (Figure 7B). The characters that contributed the most to the observed discrimination were the area of indumentum surface, the indumentum adaxial (percentage of coverage), the number of capitate hairs, the leaf surface and the average width of the leaf (Table S3).

## 3. Discussion

The AFLP analyses of *T. montanum* s.l. from the Balkan Peninsula revealed that the individuals from the 51 populations are divided into two primary genetic clusters: cluster B (*T. montanum* s. str.), which is geographically restricted to the extreme north-west of the Balkan Peninsula, and cluster A (*T. montanum* s.l.), which is distributed across the rest of the Balkan Peninsula. Cluster A is then further differentiated into four genetic subclusters, which are also clearly delimited geographically on the NW-SE and W-E transects (Figure 2 and Figure 4). A similar spatial pattern of genetic diversification has also been observed in other species on the Balkan Peninsula. For example, nearly identical cases in which populations from the northwestern part of the Balkan Peninsula exhibit marked genetic differentiation from other Balkan populations have been documented in the *Campanula pyramidalis* L. complex [20], *Edraianthus tenuifolius* (A.DC.) A.DC. [21], *Viola suavis* M.Bieb. [22], *Edraianthus graminifolius* (L.) A.DC. ex Meisn. [23], and *Salvia officinalis* L. [24]. Furthermore, similar phylogeographic patterns have been documented in numerous other studied groups in relation to other parts of the Balkan Peninsula (e.g., the *Sesleria rigida* Heuff. ex Rchb. complex—Kuzmanović et al. [25]; *Campanula* L. spp.—Lakušić et al. [20], Škondrić et al. [26], Janković et al. [27]; *Silene saxifraga* L. group—Đurović et al. [28]; *Alyssum montanum* L.—*A. repens* Baumg.—Španiel et al. [29]; *Cymbalaria* Hill. genus—*Carnicero* et al. [30], etc.).

In contrast to many other cases where genetic diversification is accompanied by significant morphological diversification, a notable discrepancy between the patterns of genetic and morphological diversification was revealed in the populations of *T. montanum* s.l. in the Balkan Peninsula. Specifically, there is no clear relationship between the morphological groups and the genetic clusters, neither at the level of the primary genetic clusters (A and B) nor at the level of the four subclusters identified by K-means clustering. In addition, it has been found that certain groups of genetically similar populations show morphological uniformity, while there are a considerable number of populations belonging to one morphological group but are assigned to different genetic clusters. For example, the phylogenetic network revealed the presence of three genetically distinct population groups within the Dinaric mountain system. These groups are separated by a bootstrap support value of over 50%, although they all belong to the same morphological group “montanum”. A similar case showed that the populations from the same mountain system, the Rhodope massif, were located in the same part of the phylogenetic network and were also assigned to the same morphological group “montanum”. On the other hand, an analysis of genetic data revealed a certain degree of genetic similarity between populations from the Scardo-Pindhic and Carpatho-Balkan mountain ranges. However, both groups encompass populations from diverse morphological groups, exhibiting considerable morphological variability.

In addition, it is also important to note that the most widespread morphological group “montanum” is genetically the most diverse, with different populations of this morpho-group being distributed in each mountain system and classified in all four K-means subclusters. It is worth noting the presence of multiple morpho-groups in the populations of the Scardo-Pindhic and the Carpatho-Balkan mountain systems. Individuals of morpho-group “montanum” show a close genetic relationship with other morphological groups, including “luteolum” and “helianthemoides” in the southern Balkans and “pannonicum” and “skorpilii” in the eastern and north-eastern parts of the Balkans.

The distribution of genetic subclusters (K1 to K4) is largely spatially separated and corresponds to their position on the phylogenetic network. However, populations with mixed genetic structures can be observed in the contact zones between mountain massifs, i.e., at the boundaries of the distribution of genetic subclusters. The observed genetic structure indicates that, in the course of the complex geological history of the Balkan Peninsula, there has been an exchange of genetic material between populations from different mountain systems and genetic clusters. This has subsequently slowed down the process of speciation in this region.

It should also be mentioned that the populations P06 MA-Kanjon Matke and P42 AL-Ostrovica, which could not be assigned to any morphological group due to their transitional morphological characteristics [9], also exhibit transitional genetic character, as they are situated at the interface between several genetic groups.

**Figure 8:**
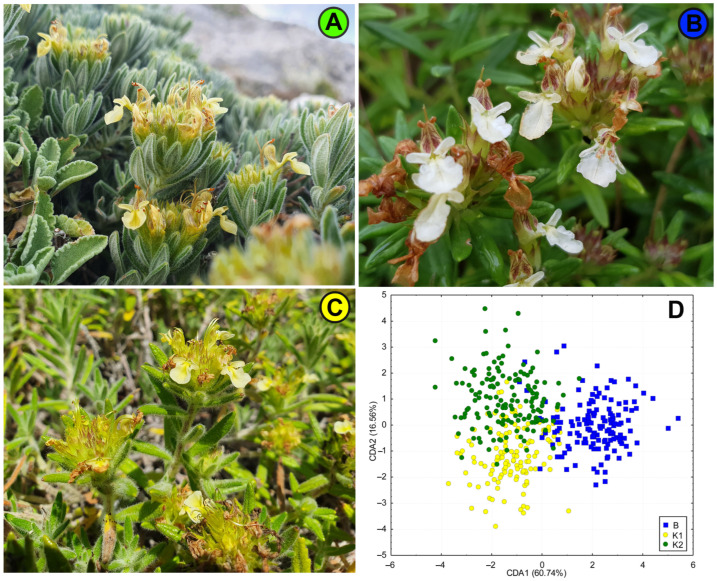
CDA scatterplot including three peripheral groups of *T. montanum* populations classified into a priori groups identified as genetic cluster B and genetic subclusters K1 and K2. (**A**)—Plant from P02 GR-Dirfi (subcluster K2), (**B**)—plant from P19 GR-Žumberak (cluster B), (**C**)—plant from P48 SR-Vratna Canyon (subcluster K1), and (**D**)—simplified CDA scatterplot. The colors of the labels on the pictures correspond to the symbols on the scatterplot. (Photos M. Zbiljić.)

It is important to emphasize that the contact populations with mixed genetic structure (populations P33, P31 and P32) have a similar morphology and belong to the same morphological group “montanum” as the geographically nearby populations. A remarkable discrepancy between genotypic and morphological diversification exists in the eastern part of the Balkan Peninsula, where genetically close populations (P35 SR-Rtanj, P51 BU-Sliven and P48 SR-Vratna Canyon) belong to different morphological groups (P35 SR-Rtanj = morphological group “montanum”, P51 BU-Sliven = “skorpili”, P48 SR-Vratna Canyon = “pannonicum”). The morphologically distinct populations P49 GR-Parnassus and P50 AL-Skadar (Figure 6) also show no genetic distinctiveness. There are also genetic and morphological divergences in the northern part of Scardo-Pindhic, where populations P01 MA-Ohrid, P10 AL-Gjergjevice and P07 GR-Malakasi are completely classified into subclaster K2, but belong to a different morphological groups (“helianthemoides”, “montanum” and “luteolum”). Although there is no overlap between the morphological and genetic diversification patterns of *T. montanum* s.l. in the Balkan Peninsula, a general correspondence can be observed between the morphological and genetic groups identified in the peripheral parts of the study area. This could indicate one of the possible evolutionary scenarios that took place in this complex in the Balkan Peninsula. Indeed, populations P19 HR-Fužine and P21 HR-Žumberak from genetic cluster B (NW Dinarides), population SR-Vratna Canyon from subcluster K1 (Southern Carpathians) and populations P02 GR-Dirfi and P49 GR-Parnassus from subcluster K2 (southern Scardo-Pindhic Massif) represent both morphologically [9] (Figure 5, Figure 7B and Figure 8) and genetically (Figure 1, Figure 3 and Figure 4) well-diversified groups. These populations could be those from which other identified morphological and genetic groups evolved during the evolutionary history of this complex in the Balkan Peninsula. The substantial discrepancy between the patterns of morphological and genetic diversification in *T. montanum* s.l. on the Balkan Peninsula can primarily be attributed to the reproductive biology of this plant group and the highly complex geological history of the Balkan Peninsula. The status of reproductive barriers, i.e., the potential for hybridization, introgression and gene flow between closely related taxa, plays a crucial role in the evolution and phylogeographic structure of any plant group. In species with weak reproductive barriers, the processes of hybridization and introgression result in the gene flow between different taxa [31,32]. This leads to a reduction in the morphological differences between the taxa and a blurring of the taxonomic boundaries in certain parts of their range [33,34]. Previous studies have demonstrated that the representatives of the Polium group (*T. montanum*, *T. polium* L. and *T. capitatum*) are very young species [35]. Additionally, hybridization between these species is a common event, as evidenced by microsatellite studies in sympatric populations demonstrating gene flow between the two closely related species [31,32]. This also suggests that reproductive barriers are very weak in *T. montanum* s.l., thereby suggesting that hybridization and introgression between morphological groups and genetic groups represent a significant contributing factor to the observed genetic and morphological patterns.

During the glacial periods, the Balkan Peninsula served as an important refuge, with numerous smaller local refuges where certain populations preserved their unique genotypes. The repeated processes of retreat and recolonization triggered by the glacial cycles led to secondary contacts between previously isolated phylogenetic lineages [3]. These processes contribute to the genetic structure observed in the present day. A notable example of this phenomenon is the divergence of the P2-Dirfi and P49-Parnassus populations, which were likely isolated over time, resulting in a partial genetic differentiation and, consequently, a morphological differentiation into two distinct morphological groups: the population of Dirfi, “helianthemoides”, and the Parnassus populations, “parnassicum”. The dispersal of these populations in an east–west direction and towards the north may have been facilitated by ecological changes over time. This dispersal resulted in contact between the populations belonging to other phylogenetic lineages or morphological groups. This contact facilitated the exchange of genetic material and the mixing of morphotypes, resulting in the differentiation of these two populations from each other while also exhibiting shared traits with populations from the northern regions of present-day Greece. Thus, the Parnassus population exhibits morphological similarities to the qualitative characteristics of the morphological group “luteolum”. In contrast, the Dirfi population exhibits morphological similarities to the morphological group “helianthemoides” from the Greek mainland, but also exhibits a number of distinctive morphological features [9,36]. In light of the absence of reproductive boundaries between groups within this complex, it can be posited that new populations of hybrid swarms emerge at each contact between more or less diversified genotypes or phenotypes. Such events undoubtedly occurred both before and during the glaciation period, as well as in the present day, not only within *Teucrium montanum sensu lato*, but also between related taxa from section Polium [31,32]. As the distribution range of these morphological and genetic groups extends beyond the borders of the Balkan Peninsula, it is necessary to apply the recently developed molecular SSR markers [31] to a broader sample, including areas where typical *T. montanum* thrive, in order to resolve and revise the taxonomic concept of the broadly defined species *Teucrium montanum*.

## 4. Materials and Methods

### 4.1. Plant Material

Analyses were based on field-collected leaf material of 4 to 13 individuals per population, and a total of 57 populations (Table S1). Leaf material for molecular studies was desiccated in silica gel. For morpho-anatomical analyses, between 6 and 21 individuals were collected from each population, and the samples were fixed in the field in a mixture of glycerol and 50% ethanol (1:1). Voucher specimens were deposited at the Herbarium of the University of Belgrade (BEOU).

### 4.2. DNA Isolation and AFLP Analysis

Total genomic DNA was extracted from c. 10 mg of dried tissue according to the procedure described in the instructions for use of the DNA extraction kit “NucleoSpin Plant II” (Macherey-Nagel, GmbH & Co. KG, Düren, Germany).

The AFLP method was performed according to a modified original protocol [37]. Four primer combinations and four fluorescent dyes were used for selective amplification: VIC-EcoRI-ACG + Tru1I-CGA, NED-EcoRI-AGA + Tru1I-CGA, FAM-EcoRI-ACA + Tru1I-CGA, and PET-EcoRI-ACC + Tru1I-CGA. The selective amplification products were detected using an ABI 3730XL analyzer (Applied Biosystems) and GeneMapper 5.0 software (Applied Biosystems).

**Intrapopulation diversity**—The AFLP fragments obtained were scored as either present (1) or absent (0). Intrapopulation diversity analysis included calculation of the proportion of polymorphic loci (%P), number of unique alleles (*N_pr_*) and frequency-down-weighted marker values (DW values) [38] using the AFLPdat v. 16.05.2007 R package [39]. The Shannon index (*I*) was calculated as *I = −*Σ *(p_i_ log*2 *p_i_)*, where *p_i_* is the phenotypic frequency [40,41]. Expected heterozygosity (*H_E_*) was estimated using a Bayesian approach [42] assuming Hardy–Weinberg equilibrium (*F_is_* = 0), as implemented in AFLP-Surv v. 1.085 [43].

**Differentiation and genetic structure of populations**—The standard genetic distance between pairs of populations was calculated according to Nei [44] using the AFLP-Surv v. 1.085 software. A neighbor-joining tree was created with the program “NEIGHBOR” from the PHYLIP package v. 3.6 [45]. To calculate the bootstrap values in the programs “NEIGHBOR” and “CONSENSE” (PHYLIP), one thousand pseudoreplicates were generated with AFLP-Surv v. 1.085. The tree was rooted with the *T. capitatum* population as the outgroup. To create a neighbor-net diagram of the populations, Nei’s distance matrix between populations [46] was used with the software SplitsTree4 v. 12.3 [47].

The genetic population structure was estimated with BAPS v. 6.0 [48] with and without the outgroup (*T. capitatum*). The maximum number of clusters (K) was set to 20 and the analysis was repeated 30 times. Population mixture analysis [49] was performed with the default settings. In order to ascertain the fine relationships between populations within genetic cluster A, a non-hierarchical *K*-means clustering algorithm was applied exclusively to the populations belonging to genetic cluster A. Non-hierarchical *K*-means clustering [50] was performed using a script of Arrigo et al. [51] in RStudio v.1.0.143 (R Studio Team [52] 2016, R-3.3.1). A total of 50 000 independent runs were performed (i.e., starting from random points) for each assumed value for K clusters ranging from 2 to 10. To select the best number of groups, the strategy proposed by Evanno, Regnaut and Goudet [53] was used, and the proportions of individuals assigned to *K*-means groups (within populations) were displayed on a map created in ArcGIS v.10.0 (Esri, Redlands, CA, USA). Finally, a neighbour-joining tree based on Jaccard distances among individuals was produced using SplitsTree4 v. 12.3 [47] for the populations belonging to genetic cluster A. The identified subclusters were marked with lines indicating the belonging of individuals to four clusters inferred by non-hierarchical *K*-means clustering.

### 4.3. Morpho-Anatomical Analyses

The anatomical analyses of the leaves were performed using slides prepared according to the standard protocol for light microscopy [11]. The cross-sections of the leaves were prepared using a Reichert sliding microtome with a section thickness of 26 µm and 65 µm. A total of 23 anatomical features were measured: 9 from leaf cross-sections with a thickness of 65 µm and 14 from cross-sections with a thickness of 26 µm.

Two leaves, five bracts, one stem and two calyxes were analyzed per individual. The leaves were selected from the middle section of the side shoots, while the stem used for morphometric analysis was taken from a side shoot creeping on the ground to ensure that the measured stem length corresponded to the height of the individual. A total of 22 morphometric characters were measured, including 2 ratio traits and 20 quantitative characters. The detailed methodology and measurement scheme can be found in Zbiljić et al. [9].

The multivariate analyses were performed at two levels. First, principal component analysis (PCA) was employed to describe the overall morphological variability and to obtain an overall structure of the morphological differences between genetic clusters A and B. For PCA, 26 of the 45 measured morphological characters were used based on the results of the analysis of variance (ANOVA) and Spearman correlation matrix, with highly correlated characters and those that did not contribute to variability excluded. In the second step, a canonical discriminant analysis (CDA) was performed to test the morphological separation between the subclusters defined by non-hierarchical K-means clustering. Non-hierarchical K-means clustering was employed only for the populations that had been previously categorized into genetic cluster A in BAPS analysis (K1, K2, K3 and K4). The multivariate statistical analyses were performed using Statistica v.8.0 [54] and Past 4.17 [55].

## Data Availability

The data presented in this study are available on request from all authors.

## Supplementary Materials

The following supporting information can be downloaded at https://www.mdpi.com/article/10.3390/plants13243596/s1, Table S1. Parameters of the genetic structure of the analyzed populations, classification of populations into genetic groups obtained based on the analysis of genetic clusters obtained by BAPS analysis with an outgroup (BAPS), and classification of populations into genetic clusters obtained based on non-hierarchical *K*-means clustering (K-means). Abbreviations: MA—North Macedonia, GR—Greece, AL—Albania, HR—Croatia, BH—Bosnia and Herzegovina, CG—Montenegro, SR– Serbia, RU—Romania, and BU– Bulgaria. Morphological groups according to Zbiljić et al. [9]: hel—“helianthemoides“, lut—“luteolum“, mon—“montanum“, pan—“pannonicum“, par—“parnassiucum“, cap—“capitatum“, ska—“skadarensis“, sko—“skorpili“. Genetic parameters: n—number of individuals per population, %P—proportion of polymorphic bands, *N_pr_*—number of unique alleles, *I*—Shannon’s information index, *H_E_*—expected heterozygosity, MG—morphological groups, BAPS—cluster BAPS, and K-means—subcluster (K-means). Admixed populations identified by *K*-means clustering are marked with asterisks (*) in populations column. Table S2. Loadings of morphological characters on the first two axes of the principal component analysis (PCA) within *T. montanum sensu lato* on the Balkan Peninsula. The characters that contribute the most to the observed variability are highlighted in bold and marked in red. Table S3. Discriminant function analysis based on 31 morphological characters for four K subclusters (K1, K2, K3 and K4) derived by non-hierarchical K-means clustering.
