# Supplementary material for: Patterns of Genetic and Morphological Variability of Teucrium montanum sensu lato (Lamiaceae) on the Balkan Peninsula
Source: Plants (Basel). 2024 Dec 23;13(24):3596. doi: 10.3390/plants13243596 (PMC11679216; doi:10.3390/plants13243596)
Supplement: Supplementary file 1 [file plants-13-03596-s001.zip › plants-3320430-supplementary.pdf]

Supplementary material:

Table S1. Parameters of the genetic structure of the analyzed populations, classification of populations into genetic groups obtained based on the analysis of genetic clusters obtained by BAPS analysis with an outgroup (**BAPS**), and classification of populations into genetic clusters obtained based on non-hierarchical *K*-means clustering (**K means**). Abrevation: MA – North Macedonia, GR – Greece, AL – Albania, HR – Croatia, BH – Bosnia and Herzegovina, CG – Montenegro, SR– Serbia, RU – Romania, BU– Bulgaria; Morphological groups according to Zbiljić et al [9] – hel: "helianthemoides", lut: "luteolum", mon: "montanum", pan: "pannonicum", par: "parnassiucum", cap: "capitatum", ska: "skadarensis", sko: "skorpili"; Genetic parameters: **n** – number of individuals per population, **%P** – proportion of polymorphic bands, N_{pr} – number of unique alleles, ***I*** – Shannon's information index, H_E – expected heterozygosity, **MG** – morphological groups, **BAPS** – cluster BAPS, **K means** – subcluster (K-means). Admixed populations identified by *K*-means clustering are marked with asterisks (*) in Populations column.

Code	Populations	Lat (N)	Long (E)	Voucher number	n	%P	N_{pr}	*I*	H_E	MG	BAPS	K means
P01	MA-Ohrid	40.954	20.813	BEOU - 54036	5	0.171	2	0.140	0.08	hel	A	K2
P02	GR-Dirfi	38.619	23.849	BEOU - 69457	5	0.124	0	0.101	0.066	hel	A	K2
P03	GR-Ossa *	39.787	22.665	BEOU - 46638	5	0.165	1	0.128	0.075	hel	A	K2/K3
P04	GR-Olimpys	40.102	22.439	BEOU - 46638	5	0.161	0	0.132	0.078	hel	A	K2
P05	GR-Askion	40.369	21.557	BEOU - 67659	5	0.147	0	0.118	0.070	hel	A	K2
P06	MA-Matka Canyon *	41.947	21.302	BEOU - 54035	5	0.127	1	0.100	0.065	hel/pan	A	K2/K1
P07	GR-Malakasi	39.799	21.23	BEOU - 67663	5	0.153	0	0.122	0.069	lut	A	K2
P08	GR-Smolikas *	40.07	20.838	BEOU - 67661	5	0.157	1	0.129	0.075	lut	A	K2/K3
P09	AL-Tomorit *	40.58	20.15	BEOU - 69458	6	0.214	0	0.159	0.078	lut/mon	A	K2/K3
P10	AL-Gjergjevice	40.585	20.579	BEOU - 54037	5	0.155	0	0.127	0.074	mon	A	K2
P11	HR-Biokovo	43.345	17.053	BEOU - 54030	5	0.157	1	0.124	0.074	mon	B	
P12	HR-Bisko, Trilj	43.579	16.696	BEOU - 54028	5	0.202	0	0.159	0.089	mon	B	
P13	HR-Murter	43.79	15.615	BEOU - 54026	5	0.188	4	0.153	0.088	mon	B	
P14	HR-Gračac	44.362	15.867	BEOU - 54025	5	0.180	0	0.148	0.087	mon	B	
P15	HR-Oštarije	44.528	15.138	BEOU - 54024	5	0.135	0	0.110	0.069	mon	B	
P16	HR-Premantura	44.806	13.877	BEOU - 54022	5	0.155	0	0.129	0.079	mon	B	
P17	HR-Cres	44.85	14.42	BEOU	5	0.153	0	0.126	0.076	mon	B	
P18	HR-Sv. Jelena	45.015	14.89	BEOU - 54023	5	0.184	0	0.150	0.084	mon	B	
P19	HR-Fužine	45.332	14.689	BEOU - 54020	5	0.157	0	0.131	0.077	mon	B	
P20	HR-Lanišće	45.492	14.03	BEOU - 54021	5	0.255	0	0.199	0.093	mon	B	
P21	HR-Žumberak	45.72	15.45	BEOU - 54019	5	0.122	0	0.099	0.062	mon	B	
P22	BH-Trebinje	42.7	18.362	BEOU - 54033	5	0.202	1	0.163	0.088	mon	A	K3
P23	BH-Korita	43.047	18.49	BEOU - 54034	5	0.180	0	0.142	0.081	mon	A	K3
P24	BH-Vlašić	44.274	17.614	BEOU - 68400	5	0.139	0	0.112	0.071	mon	A	K4
P25	BH-Vrbas Gorge	44.459	17.16	BEOU - 68398	5	0.135	0	0.113	0.074	mon	A	K4
P26	BH-Pribinić	44.61	17.702	BEOU - 68401	5	0.155	0	0.128	0.077	mon	A	K4
P27	BH-Ozren	44.665	18.202	BEOU - 68397	5	0.149	0	0.124	0.079	mon	A	K4
P28	CG-Lovćen	42.38	18.85	BEOU - 54019	5	0.137	0	0.111	0.067	mon	A	K3
P29	CG-Orjen	42.562	18.535	BEOU - 54017	5	0.139	0	0.112	0.067	mon	A	K3
P30	CG-Bjelasica*	42.84	19.71	BEOU - 69148	5	0.155	0	0.126	0.076	mon	A	K3/K4
P31	SR-Brezovica*	42.228	21.009	BEOU - 54040	5	0.159	0	0.128	0.075	mon	A	K1/K2/K3

Code	Populations	Lat (N)	Long (E)	Voucher number	n	%P	N_{pr}	*I*	H_E	MG	BAPS	K means
P32	SR-Šarplanina, Pribeg *	42.185	21.048	BEOU - 54039	5	0.192	1	0.155	0.084	mon	A	K1/K2/K3
P33	SR-Vlajkovci*	43.338	20.943	BEOU - 46883	5	0.151	0	0.122	0.074	mon	A	K1/K2/K4
P34	SR-Sićevo	43.338	22.078	BEOU - 46878	5	0.133	0	0.109	0.069	mon	A	K1
P35	SR-Rtanj	43.74	21.863	BEOU - 46882	5	0.155	0	0.124	0.073	mon	A	K1
P36	SR-Zaovine	43.86	19.41	BEOU - 46618	5	0.165	0	0.134	0.079	mon	A	K4
P37	SR-Orovica	43.914	20.129	BEOU - 54013	5	0.104	2	0.085	0.059	mon	A	K4
P38	SR-Trešnjica	44.148	19.557	BEOU - 46643	5	0.147	0	0.114	0.073	mon	A	K4
P39	RU-Nera Canyon *	44.903	21.736	BEOU - 54012	5	0.157	2	0.127	0.075	mon	A	K1/K4
P40	BU-Pirin	41.766	23.43	BEOU - 68403	5	0.135	0	0.113	0.071	mon	A	K1
P41	BU-Golo brdo	42.48	23.084	BEOU - 68402	5	0.149	0	0.122	0.074	mon	A	K1
P42	AL-Ostrovica	40.568	20.466	BEOU - 54038	5	0.186	1	0.154	0.082	mon	A	K2
P43	AL-Deja*	41.686	20.138	BEOU - 47262	5	0.149	0	0.120	0.069	mon	A	K2/K3
P44	GR-Kryoneri	41.042	24.372	BEOU - 67667	5	0.135	0	0.108	0.066	mon	A	K1
P45	GR-Chianochori	41.142	23.646	BEOU - 67665	5	0.114	1	0.093	0.061	mon	A	K1
P46	GR-Falakro	41.29	24.027	BEOU - 67666	5	0.149	0	0.119	0.070	mon	A	K1
P47	SR-Gornjak	44.273	21.534	BEOU - 46880	5	0.157	1	0.122	0.074	pan	A	K1
P48	SR-Vratna Canyon	44.383	22.336	BEOU - 69147	5	0.161	0	0.133	0.079	pan	A	K1
P49	GR-Parnassus	38.496	22.651	BEOU - 69456	4	0.129	0	0.110	0.073	par	A	K2
P50	AL-Skadar*	42.008	19.683	BEOU - 46619	5	0.194	0	0.156	0.079	ska	A	K3/K2
P51	BU-Sliven	42.74	26.313	BEOU - 68404	5	0.151	0	0.126	0.076	sko	A	K1
P52	SR-Sićevo , *T. capitatum*	43.338	22.078	BEOU - 70901	5	0.206	7	0.170	0.105	cap	C	

Table S2. Loadings of morphological characters on the first two axes of the principal component analysis (PCA) within *T. montanum sensu lato* on the Balkan Peninsula. The characters that contribute the most to the observed variability are highlighted in bold and marked in red.

Characters	Acronyms	Factor 1	Factor 2
Leaf curvature	L_Curv	-0.523849	0.533225
Indumentum adaxial (percentage of coverage)	C_In-ad	0.271931	0.353592
Indumentum abaxial (percentage of coverage)	C_In-ab	0.023488	-0.113117
Width of the leaf	W_L	-0.606323	0.505128
Radius of the oil-containing cell	R_OC	-0.183563	-0.082714
Number of oil-containing cell	No_OC	-0.275016	0.154704
Number of oil-containing cell	No_CH	-0.252397	0.334097
Thickness of palisade tissue - adaxial	T_Pal-ad	-0.086099	-0.074086
Number of palisade layers	No_Pal_l	0.172140	0.072169
Leaf surface	**L_S**	**-0.939205**	0.047156
Average width of leaf	**Avg_w_l**	**-0.830862**	0.423688
Leaf base width	L_B_L	-0.531448	0.122618
Leaf length (µm)	**L_L**	**-0.853841**	-0.355638
Ration between the widest part of leaf and leaf length	W_L/L_L	-0.088561	**0.870425**
Distance between leaf base and the widest part of the leaf (Distance 1)	**D1**	**-0.780477**	-0.272528
Number of teeth on leaf margine (µm)	L_T	-0.312195	0.286666
Bract length	B_L	-0.621139	-0.184048
Stem height	**S_H**	**-0.771942**	-0.226169
Average length of first three internodes	Avg_L_F_I	-0.674341	-0.226585
Average length of last three internodes	Avg_L_L_I	-0.608827	-0.199914
Percentage of yellow in corolla	Yellow_%	0.205266	0.341430
Distance between calyx base and tooth base	D_Cal_b_T_b	-0.456584	0.102524
Distance between tooth base and narrow part of tooth	D_T_b_T_t	-0.461222	0.106179
Length of the narrow part of tooth	L_n_T	-0.218262	-0.319163
Number of flowers in terminal inflorescence	No_F_I	-0.356328	-0.250650
	Expl.Var	6.670046	2.547119
	Prp.Totl	0.266802	0.101885

Table S3. Discriminant function analysis based on 31 morphological characters for four K subclusters (K1, K2, K3 and K4) derived by non-hierarchical K-Means clustering.

Characters	Acronyms	F-remove (3.406)	p-level
Number of capitate hairs	No_CH	19.37506	0.0000
Leaf surface	L_S	14.48283	0.0000
Indumentum adaxial (percentage of coverage)	C_In-ad	14.36039	0.0000
Average width of leaf	Avg_w_l	11.77360	0.0000
Leaf curvature	L_Curv	9.79271	0.0000
Percentage of yellow in corolla	Yellow_%	9.09099	0.0000
Bract length	B_L	8.97214	0.0000
Ration between the widest part of leaf and leaf length	W_L/L_L	8.93192	0.0000
Area of indumentum surface / 1000	A_In	8.73090	0.0000
Number of teeth on leaf margine	L_T	8.17830	0.0000
Average length of first three internodes	Avg_L_F_I	8.17365	0.0000
Thickness of adaxial epidermal cells	T_Epi-ad	7.07061	0.0001
Distance between calyx base and tooth base	D_Cal_b_T_b	6.70682	0.0002
Area of leaf surface / 1000	A_L	6.17564	0.0004
Radius of the oil-containing cell	R_OC	6.00699	0.0005
Radius peltate hairs	R_PH	5.84727	0.0006
Stem height	S_H	5.44863	0.0011
Leaf center curvature	R_LC	4.85035	0.0025
Width of the leaf	W_L	3.97972	0.0081
Distance between tooth base and narrow part of tooth	D_T_b_T_t	3.91348	0.0089
Indumentum abaxial (percentage of coverage)	C_In-ab	3.64386	0.0128
Average width of bract	Avg_w_b	3.60213	0.0136
Number of flowers in terminal inflorescence	No_F_I	3.32227	0.0198
Ration between distance D1 and leaf length	D1/L_L	2.80861	0.0393
Central nerve	R_CN	2.56700	0.0541
Length of the narrow part of tooth	L_n_T	2.17686	0.0901
Thickness of abaxial epidermal cells	T_Epi-ab	1.24939	0.2914
Thickness of the leaf	T_L	1.15185	0.3279
Average length of last three internodes	Avg_L_L_I	1.00323	0.3912
Number of oil-containing cell	No_OC	0.41520	0.7421
Leaf base width	L_B_L	0.18351	0.9075
